# Supplementary material for: Synthesis and Structure of Novel Copper(II) Complexes with N,O- or N,N-Donors as Radical Scavengers and a Functional Model of the Active Sites in Metalloenzymes
Source: Int J Mol Sci. 2021 Jul 6;22(14):7286. doi: 10.3390/ijms22147286 (PMC8307904; doi:10.3390/ijms22147286)
Supplement: Supplementary file 1 [file ijms-22-07286-s001.zip › ijms-1274096-final SI.pdf]

Synthesis and structure of novel copper(II) complexes with N,O- or N,N-donors as radical scavengers and a functional model of the active sites in metalloenzymes

Joanna Masternak, Małgorzata Zienkiewicz-Machnik, Iwona Łakomska, Maciej Hodorowicz, Katarzyna Kazimierczuk, Milena Nosek, Amelia Majkowska-Młynarczyk, Joanna Wietrzyk, Barbara Barszcz

Table S1. Selected bond lengths (Å) for compounds **1** - **5**.

**1**		**2**		**3**		**4**		**5**	
Cu(1)-O(3)	1.939(2)	Cu(1)-N(1)	1.990(1)	Cu(1)-O(2)	1.923(5)	Cu(1)-N(1A)	1.909(5)	Cu(1)-N(1)	2.0083(19)
Cu(1)-N(3)	2.021(3)	Cu(1)-O(17)	2.001(1)	Cu(1)-O(2)^{i}	1.936(5)	Cu(1)-N(1A)^{i}	1.910(5)	Cu(1)-N(1)^{i}	2.0083(19)
Cu(1)-N(1)	2.023(3)	Cu(1)-O(8)	2.014(1)	Cu(1)-N(2)	1.992(7)	Cu(1)-N(1)^{i}	2.011(2)	Cu(1)-N(13)	2.0097(19)
Cu(1)-N(2)	2.037(3)	Cu(1)-O(16)	2.240(1)	Cu(1)-N(1)	2.024(6)	Cu(1)-N(1)	2.011(2)	Cu(1)-N(13)^{i}	2.0097(19)
Cu(1)-O(1)	2.295(3)	Cu(1)-O(18)	2.313(1)	Cu(1)-O(1)	2.288(6)	Cu(1)-N(3)	2.252(3)	Cu(1)-O(14)	2.4429(1)
Cu(1)-O(2)	2.426(3)	Cu(1)-N(9)	1.997(1)			Cu(1)-N(3)^{i}	2.252(3)	Cu(1)-O(14)^{i}	2.4429(1)

Symmetry transformations used to generate equivalent atoms for: (**3**) (i) -x+1, y, -z+½; (**4**) (i) -x+1½, -y+½, z; (**5**) (i) -x+1, -y, -z+1;

Table S2. Selected valence angles (°) for compounds **1** - **5**.

**1**		**2**		**3**		**4**		**5**	
O(3)-Cu(1)-N(3)	81.93(11)	N(1)-Cu(1)-N(9)	168.80(5)	O(2)-Cu(1)-O(2)^{i}	76.1(2)	N(1A)-Cu(1)-N(1A)^{i}	41.3(3)	N(1)-Cu(1)-N(1)^{i}	180.0
O(3)-Cu(1)-N(1)	175.44(11)	N(1)-Cu(1)-O(17)	93.98(5)	O(2)-Cu(1)-N(2)	92.8(2)	N(1A)^{i}-Cu(1)-N(1)	96.92(1)	N(1)-Cu(1)-N(13)	88.50(8)
N(3)-Cu(1)-N(1)	95.25(12)	N(9)-Cu(1)-O(17)	93.29(5)	O(2)^{i}-Cu(1)-N(2)	166.3(2)	N(1A)^{i}-Cu(1)-N(1)^{i}	96.48(1)	N(1)-Cu(1)-N(13)^{i}	91.50(8)
O(3)-Cu(1)-N(2)	88.44(10)	N(1)-Cu(1)-O(8)	80.91(5)	O(2)-Cu(1)-N(1)	166.3(3)	N(1)-Cu(1)-N(1)^{i}	165.67(1)	N(13)-Cu(1)-N(13)^{i}	180.0
N(3)-Cu(1)-N(2)	166.70(12)	N(9)-Cu(1)-O(8)	92.42(5)	O(2)^{i}-Cu(1)-N(1)	93.7(2)	N(1A)-Cu(1)-N(3)	114.85(2)	O(14)-Cu(1)-O(14)^{i}	180.0
N(1)-Cu(1)-N(2)	94.90(11)	O(17)-Cu(1)-O(8)	173.31(5)	N(2)-Cu(1)-N(1)	95.8(3)	N(1A)-Cu(1)-N(3)^{i}	155.46(2)	O(14)-Cu(1)-N(1)	74.821(1)
O(3)-Cu(1)-O(1)	101.65(11)	N(1)-Cu(1)-O(16)	93.86(5)	O(2)-Cu(1)-O(1)	102.0(2)	N(1)-Cu(1)-N(3)^{i}	91.94(1)	O(14)-Cu(1)-N(13)	72.458(1)
N(3)-Cu(1)-O(1)	97.47(14)	N(9)-Cu(1)-O(16)	78.14(5)	O(2)^{i}-Cu(1)-O(1)	93.6(2)	N(1)-Cu(1)-N(3)	77.81(1)	O(14)-Cu(1)-N(13)^{i}	107.542(1)
N(1)-Cu(1)-O(1)	75.09(11)	O(17)-Cu(1)-O(16)	86.12(5)	N(2)-Cu(1)-O(1)	96.7(3)	N(3)-Cu(1)-N(3)^{i}	89.43(1)	O(14)-Cu(1)-N(1)^{i}	105.179(1)
N(2)-Cu(1)-O(1)	93.43(13)	O(8)-Cu(1)-O(16)	98.47(5)	N(1)-Cu(1)-O(1)	87.6(2)				
O(3)-Cu(1)-O(2)	91.49(10)	N(1)-Cu(1)-O(18)	91.25(5)						
N(3)-Cu(1)-O(2)	96.10(11)	N(9)-Cu(1)-O(18)	97.89(5)						
N(1)-Cu(1)-O(2)	92.38(10)	O(17)-Cu(1)-O(18)	84.80(5)						
N(2)-Cu(1)-O(2)	74.91(10)	O(8)-Cu(1)-O(18)	90.96(5)						
O(1)-Cu(1)-O(2)	162.28(11)	O(16)-Cu(1)-O(18)	169.87(5)						

Symmetry transformations used to generate equivalent atoms for: (**3**) -x+1, y, -z+½; (**4**) (i) -x+1½, -y+½, z; (**5**) (i) -x+1, -y, -z+1;

Table S3. Hydrogen bonds and selected interactions for compounds **1 - 5** (Å and °).

Compound	D-H···A	(D-H) (Å)	(H···A) (Å)	(D···A) (Å)	<(DH···A) (°)
1	O(1)-H(1) ···Cl(1)^{i}	0.87	2.63	3.479(3)	165.9
	O2(1)-H(1) ···O(5)^{ii}	0.87	1.90	2.731(4)	159.1
2	C(2)-H(2) ···O(17)	0.95	2.48	3.03(2)	117.2
	C(3)-H(3) ···F(4)^{i}	0.95	2.50	3.31(2)	142.4
	C(5)-H(5) ···F(2)^{ii}	0.95	2.43	3.29(2)	150.7
	C(7)-H(7A) ···F(5)	0.99	2.63	3.32(2)	126.6
	C(10)-H(10) ···F(4)^{iii}	0.95	2.53	3.16(2)	123.8
	C(13)-H(13) ···O(18)^{iv}	0.95	2.61	3.36(2)	135.2
	C(15)-H(15A) ···F(2)	0.99	2.49	3.38(2)	149.3
	O(8)-H(8) ···F(5)^{iii}	0.84(3)	1.74(3)	2.58(1)	176(3)
	O(16)-H(16) ···F(1)	0.74(3)	1.97(3)	2.71(1)	177(3)
	O(17)-H(17A) ···F(1)^{v}	0.78(2)	1.98(2)	2.77(1)	177(2)
	O(17)-H(17A) ···F(6)^{v}	0.78(2)	2.43(2)	2.88(1)	118(2)
	O(17)-H(17B) ···F(6)^{vi}	0.85(2)	1.78(3)	2.63(1)	178(2)
	O(18)-H(18A) ···F(3)^{iii}	0.74(2)	1.95(2)	2.69(2)	178(2)
	O(18)-H(18B) ···F(4)^{vi}	0.80(3)	1.96(3)	2.76(2)	172(2)
3	C(8)-H(8) ···O(5A)	0.95	2.7173(127)	3.4609(151)	135.7
	C(9)-H(9) ···O(5)	0.95	2.4763(117)	3.3027(142)	145.1
	C(13)-H(13B) ···O(1)	0.99	2.63(7)	3.4268(111)	134.1
	C(13)-H(13B) ···O(3)^{i}	0.98	2.719(72)	3.6593(113)	158.4
	C(11)-H(11) ···O(3)^{i}	0.95	2.6185(75)	3.5055(114)	155.7
	C(1)-H(1) ···O(3)^{ii}	0.95	2.5635(74)	3.2237(107)	127.0
	C(10)-H(10) ···O(6A)^{iii}	0.95	2.3348(138)	3.0015(163)	126.8
	C(6)-H(6A) ···O(5A)^{iv}	0.99	2.5293(126)	3.2491(151)	129.3
	C(7)-H(7A) ···O(5)^{iv}	0.99	2.7414(113)	3.3224(141)	117.9
	C(7)-H(7A) ···O(6A)^{v}	0.99	2.6313(133)	3.5059(159)	147.4
	C(8)-H(8) ···O(6)^{vi}	0.95	2.2416(120)	3.0351(149)	142.3
4	C(2)-H(2)-N(3A)^{i}	0.93	2.64	3.38(2)	136.9
	C(2B)^{i}-H(2B)^{i}-N(3)	0.93	2.69	3.491(7)	145.3
	N(2)-H(2N)-O(2)^{ii}	0.98(4)	1.81(4)	2.731(4)	156(4)
	O(2)-H(2BO)-F(2A)	0.97(2)	2.06(5)	2.766(1)	129(5)
	O(2)-H(2BO)-F(2B)	0.97(2)	1.97(2)	2.930(1)	172(6)
	O(2)-H(2BO)-F(4B)	0.97(2)	2.51(6)	3.08(3)	118(4)
	O(2)-H(2AO)-F(1A)^{iii}	0.96(2)	2.01(3)	2.961(1)	171(6)
	O(2)-H(2AO)-F(4A)^{iii}	0.96(2)	2.34(4)	3.103(1)	136(4)
	O(2)-H(2AO)-F(1B)^{iii}	0.96(2)	1.76(4)	2.64(2)	150(6)
	N(2A)^{i}-H(2AN)^{i}-O(1)	1.02(2)	1.91(3)	2.746(7)	137.3(1)
5	O(15)-H(15)-O(22)^{i}	0.79(4)	2.15(4)	2.787(3)	137(4)
	C(12)-H(12)-O(23)	0.95	2.55	3.300(4)	136.1
	C(2)^{ii}-H(2)^{ii}-O(23)	0.95	2.32	3.209(4)	154.6
	O(14)-H(14)-Cl(2)	0.77(4)	2.83(4)	3.5938(19)	172(3)
	O(14)-H(14)-O(21)	0.77(4)	2.12(4)	2.833(3)	154(3)
	O(14)-H(14)-O(24)	0.77(4)	2.46(4)	3.129(3)	147(3)

Symmetry transformations used to generate equivalent atoms for: (**1**) (i) -x+½, -y+½, -z+1, (ii) x-½, y-½, z; (**2**) (i) –x+1, y+½, -z+1½, (ii) x, -y-½, z-½, (iii) –x+2, -y, -z+2, (iv) x, -y+½, z+½, (v) –x+1, -y, -z+2, (vi) x, y+1, z; (**3**) (i) x+½, y+½, -z+½, (ii) -x+1, y, -z+½, (iii) -x+1½, y-½, z, (iv) -x+1, -y+1, -z+1, (v) x, y-1, z, (vi) -x+1, -y+2, -z+1; (**4**) (i) -x+1½, -y+½, z;, (ii) -x+1½, y, z-½; (iii) x-½, -y, -z-½; (**5**) (i) -x, -y, -z;

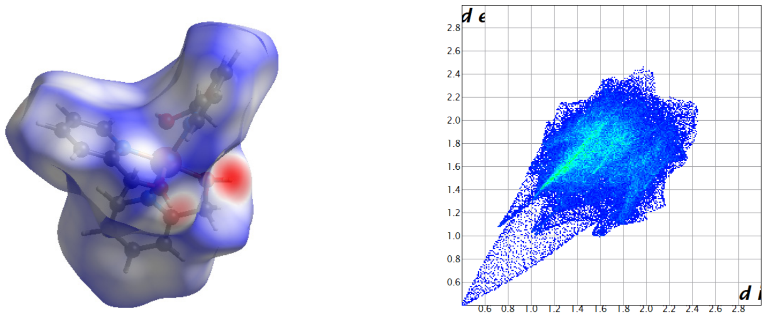

1

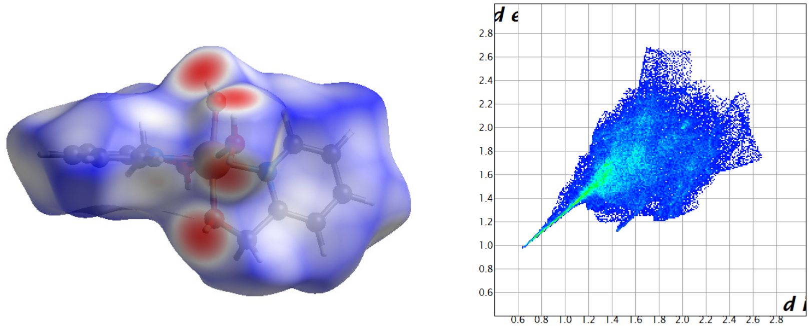

2

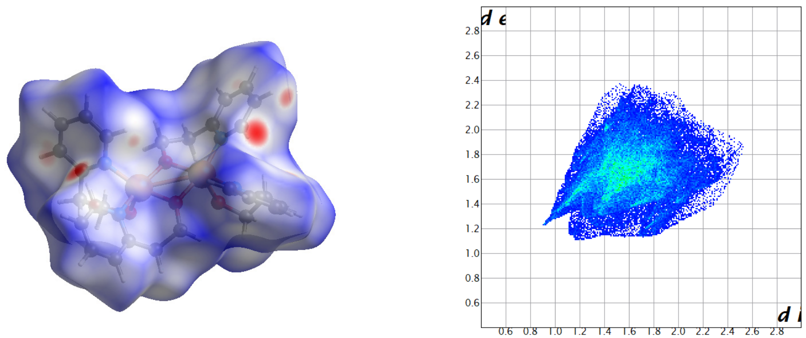

3

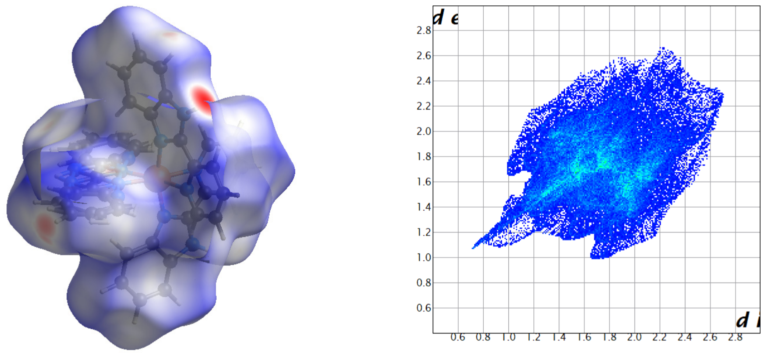

4

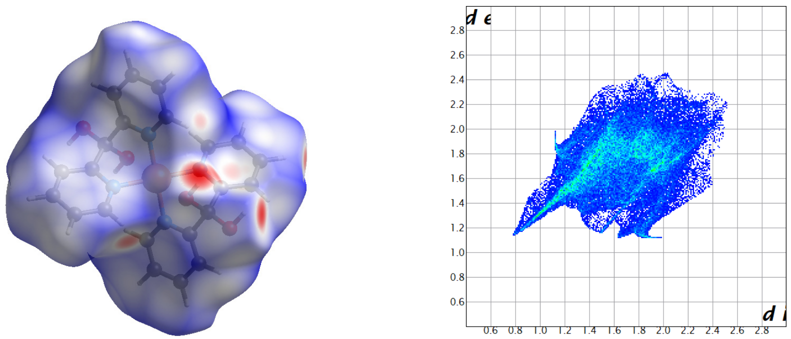

5

Figure S1. Hirshfeld surface analysis of the copper(II) complexes showing the d_{norm} and fingerprint plots.

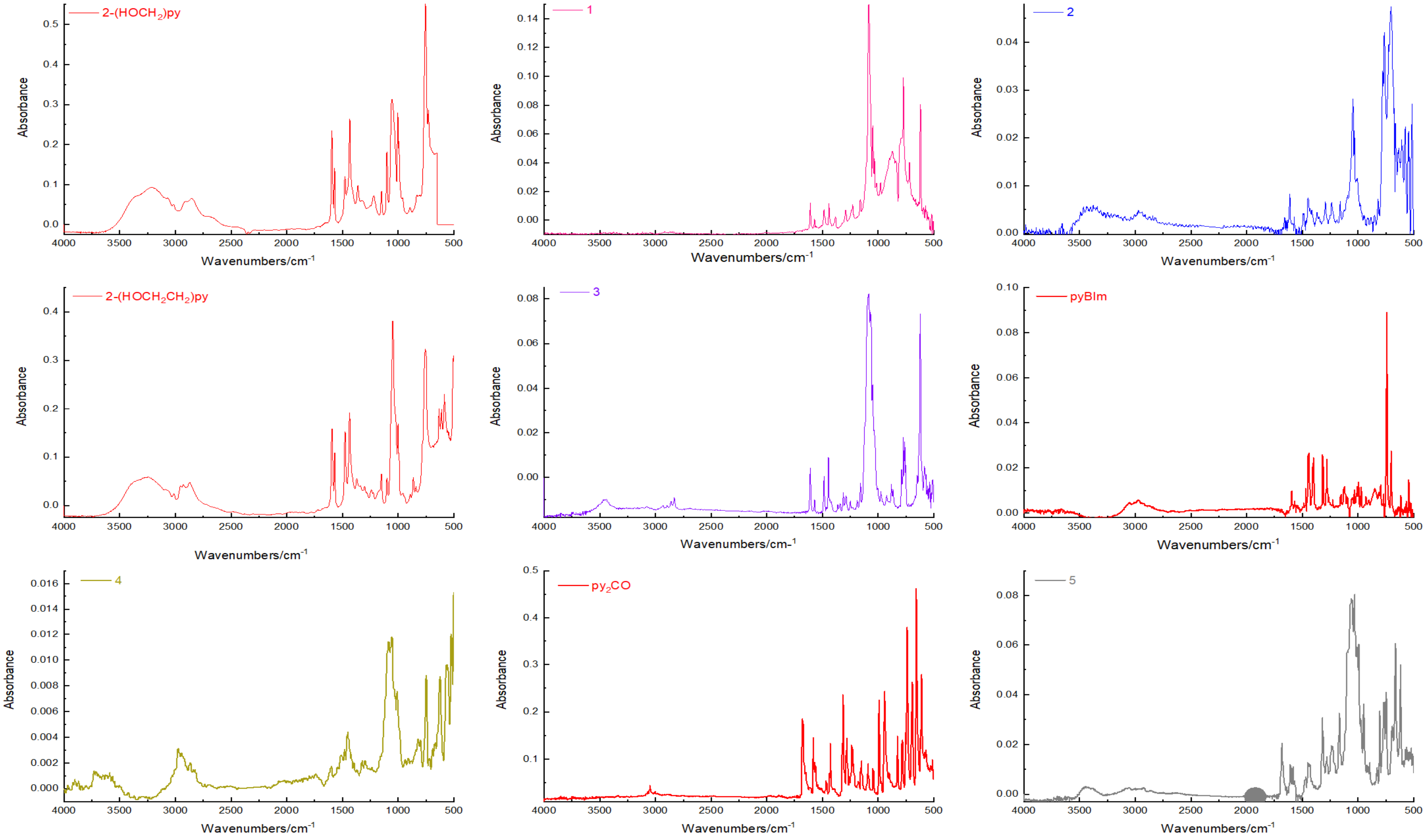

Figure S2. FTIR spectra of free ligands (red line) and copper(II) complexes.

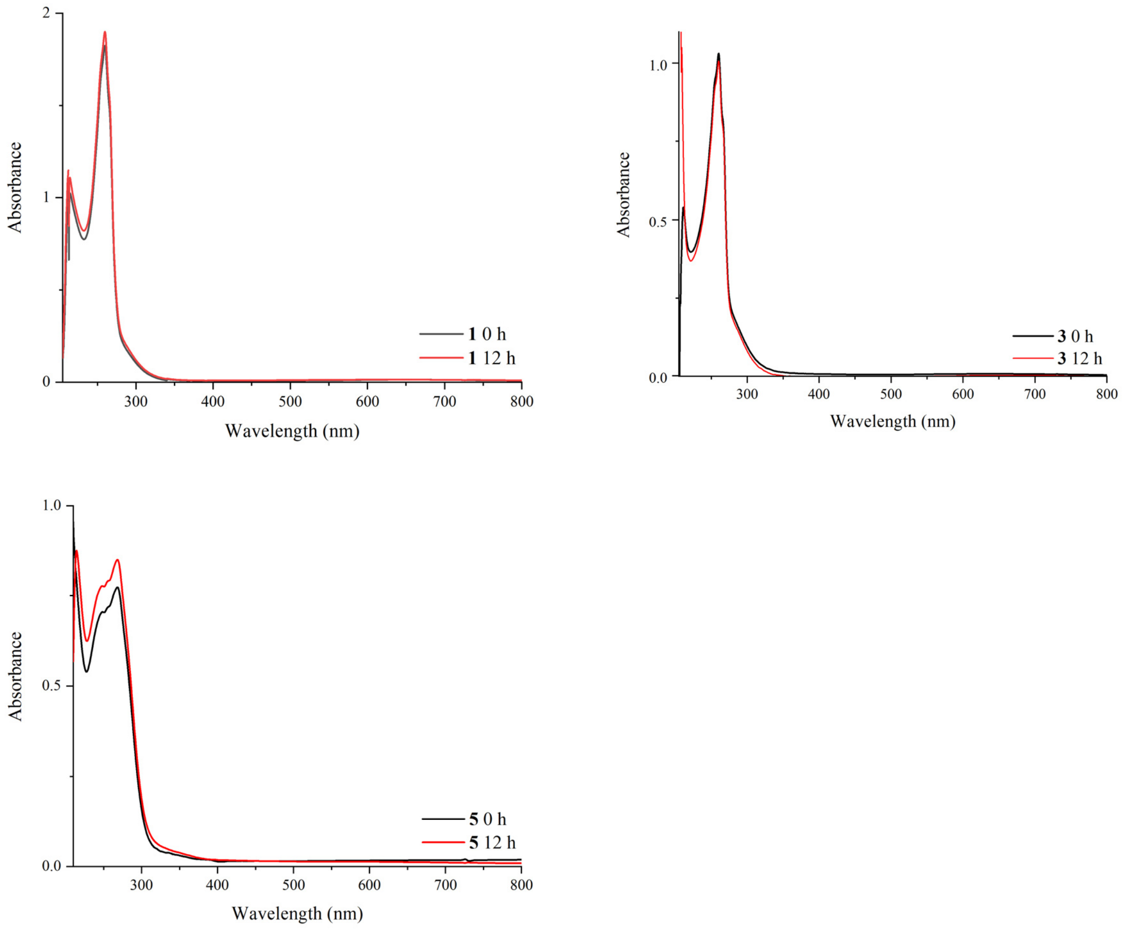

Figure S3. Selected copper complexes (**1**, **3** and **5**) stability in PBS after 0 or 12 h of incubation determined by UV-Vis spectrometry.
